# rAd5/NYVAC-B is superior to NYVAC-B/rAd5 and is dependent on rAd5 dose for neutralizing antibody responses against HIV-1

**DOI:** 10.1186/1742-4690-9-S2-P132

**Published:** 2012-09-13

**Authors:** DC Montefiori, Y Huang, S Karuna, M Allen, N Kochar, S Chappuis, J Gaillard, G Tomaras, B Graham, P Bart, G Pantaleo

**Affiliations:** 1 Duke University Medical Center, Durham, NC, USA; 2 SCHARP, Seattle, WA, USA; 3 Fred Hutchinson Cancer Research Center/HVTN, Seattle, WA, USA; 4 NIAID-NIH, Bethesda, MD, USA; 5 Lausanne University Hospital, Switzerland; 6 Vaccine Research Center, NIH, Bethesda, MD, USA

## Background

HVTN 078 is a phase 1b clinical trial of heterologous vector prime/boost vaccine regimens (NYVAC-B/rAd5 vs. rAd5/NYVAC-B) in healthy, HIV-1 uninfected, Ad5 seronegative adults. The rAd5 expressed a clade B Gag-Pol fusion protein and secreted gp140s of HIV-1 strains 92RW020 (clade A), HxB2/Bal-V3/ V1V2 (clade B) and 97ZA012 (clade C). The NYVAC-B expressed a clade B Gag-Pol-Nef polyprotein and the secreted gp120 of Bx08 (clade B). A total of 80 participants were randomized into a placebo group (P) and four treatment groups: T1, 2x NYVAC-B/1x rAd5 (10^10^); T2, 1x rAd5 (10^8^)/2x NYVAC-B; T3, 1x rAd5 (10^9^)/2x NYVAC-B; T4, 1x rAd5 (10^10^)/2x NYVAC-B.

## Methods

Binding and neutralizing antibodies were assessed at 2 weeks post-final boosting. Neutralization was assessed with tier 1 and tier 2 Env-pseudotyped viruses in TZM-bl cells, and with tier 2 Env.IMC.LucR viruses in A3R5 cells.

## Results

A dose effect for increasing anti-Env binding antibodies was seen, with higher doses of rAd5 being optimal. For neutralizing antibodies, positive response rates/median titers across the treatment groups were highest against MN.3 (69.3%/116) followed by SF162.LS (42.1%/54), BaL.26 (18.4%/15.5), MW965.26 (14.5%/31) and Bx08.16 (11.8%/19.5). Five subjects neutralized all 5 tier 1 viruses, 5 subjects neutralized 4 viruses, 7 subjects neutralized 3 viruses, 14 subjects neutralized 2 viruses (MN.3 and SF162.LS) and 18 subjects neutralized 1 virus (MN.3). Aggregate magnitude-breadth scores across the tier 1 panel were strongest for T4 followed by T3, T1 and T2. Differences were significant for T1 vs. T3 (p=0.048) and T1 vs. T4 (p=0.004). Responses against tier 2 viruses were weak and sporadic in the A3R5 assay and were nearly absent in the TZM-bl assay.

## Conclusion

A 10^10^ dose of rAd5 was superior to the two lower doses of 10^9^ and 10^8^ for both binding and neutralizing antibodies. At the highest rAd5 dose tested, rAd5/NYVAC-B was superior to NYVAC-B/rAd5 for neutralizing antibodies.

